# North–south collaboration and capacity development in global health research in low- and middle-income countries – the ARCADE projects

**DOI:** 10.3402/gha.v9.30524

**Published:** 2016-10-06

**Authors:** Salla Atkins, Sophie Marsden, Vishal Diwan, Merrick Zwarenstein, for the ARCADE consortium

**Affiliations:** 1 Department of Public Health Sciences (Global Health/IHCAR), Karolinska Institutet, Stockholm, Sweden; 2 Institute of Development Studies, Brighton, UK; 3 Department of Public Health and Environment, R.D. Gardi Medical College, Ujjain, India; 4 International Centre for Health Research, Ujjain Charitable Trust Hospital and Research Centre, Ujjain, India; 5 Department of Family Medicine, Centre for Studies in Family Medicine, Schulich School of Medicine & Dentistry, Western University, London, ON, Canada

**Keywords:** capacity building, health determinants, global health

## Abstract

**Background:**

Research capacity enhancement is needed in low- and middle-income countries (LMICs) for improved health, wellbeing, and health systems’ development. In this article, we discuss two capacity-building projects, the African/Asian Regional Capacity Development (ARCADE) in Health Systems and Services Research (HSSR) and Research on Social Determinants of Health (RSDH), implemented from 2011 to 2015. The two projects focussed on providing courses in HSSR and social determinants of health research, and on developing collaborations between universities, along with capacity in LMIC universities to manage research grant submissions, financing, and reporting. Both face-to-face and sustainable online teaching and learning resources were used in training at higher postgraduate levels (Masters and Doctoral level).

**Design:**

We collated project meeting and discussion minutes along with project periodic reports and deliverables. We extracted key outcomes from these, reflected on these in discussions, and summarised them for this paper.

**Results:**

Nearly 55 courses and modules were developed that were delivered to over 920 postgraduate students in Africa, Asia, and Europe. Junior researchers were mentored in presenting, developing, and delivering courses, and in preparing research proposals. In total, 60 collaborative funding proposals were prepared. The consortia also developed institutional capacity in research dissemination and grants management through webinars and workshops.

**Discussion:**

ARCADE HSSR and ARCADE RSDH were comprehensive programmes, focussing on developing the research skills, knowledge, and capabilities of junior researchers. One of the main strengths of these programmes was the focus on network building amongst the partner institutions, where each partner brought skills, expertise, and diverse work cultures into the consortium. Through these efforts, the projects improved both the capacity of junior researchers and the research environment in Africa, Asia, and Europe.

## Introduction

High-quality research and a sound evidence base should inform decision-making in all areas of governance and service delivery, none more so than in the field of global health (1). Increasing the capacity to carry out health research is key to global health efforts aimed at improving health services and the health of the population. However, scientific leadership is scarce in those countries most needing high-quality research evidence to inform action. This gap in capacity results in the 90/10 gap, the phenomenon that 90% of the health research is done in countries with 10% of the world's health problems (2, 3), and in a mismatch between the disease burden and the technical and human capacity for health research in low- and middle-income countries (LMICs) (4).

Nurturing local scientific leadership and research capacity is key to capacity building in LMICs (5, 6). Despite efforts to build capacity, which has increased publications originating from Africa (7), this increase in publications is small. There is also a particular lack of relevant research for decision-making, including systematic reviews, which are key to informing policymakers (8). Only 16% of policy-relevant documents in the Health Systems Evidence repository (www.healthsystemsevidence.org, intended as a free repository for evidence for supporting and strengthening health systems) had a LMIC focus in 2013 (9). These gaps are detrimental to local health systems and their policymakers, who need rigorous, summarised, local, and international evidence of impact that can be considered alongside evidence on local modifying factors such as needs, values, costs, and availability of resources (10).

There are a number of complex and interconnected factors that affect the ability of, and opportunities for, southern authors to produce research that will inform and influence health policy and practice at both national and global level. Langer et al. (11) identified five contributing factors: poor research production (in terms of both quantity and quality) and a critical lack of support for research development activities (including infrastructure and incentives), poor preparation of manuscripts, poor access to scientific journals, poor participation in publication related decision-making processes, and a bias of journals against LMIC authors.

In order to address some of these factors, several large capacity-building initiatives were funded through the European Union's 7th framework programme (2011–2015). These included south–north consortia, such as INDEPTH training and research centres of excellence (INTREC) (12), Consortium for Health Systems Policy Analysis in Africa (CHEPSAA) (13), and the African/Asian Regional Capacity Development (ARCADE) in Health Systems and Services Research (HSSR) and Research on Social Determinants of Health (RSDH). (see www.arcade-project.org for the project description). The ARCADE projects that are the focus of this paper developed teaching resources; trained students; and built institutional capacity in communications, grant writing, and grant management. Another capacity-building project, INTREC, combined online training on RSDH with workshops on mixed research methods, while CHEPSAA focussed on networking, short course development, and institutional capacity in communications and IT resources. Each took a slightly different lens to capacity building, with slightly different methods, resulting in outcomes that are not directly comparable.

In this article, we describe ARCADE HSSR and ARCADE RSDH and their outcomes in more detail. The consortia focussed on two key areas in research for global health (14): HSSR in Africa; and RSDH in Asia. They aimed to increase postgraduate students’ research capacity in Africa and Asia, with a stronger focus on PhD-level students in Africa. The consortia employed e-learning principles (15), particularly blended learning (16), which is considered more participatory than fully online courses (17) such as Massive Open Online Courses (MOOCs). We describe a number of activities that contributed to both student and institutional capacity.

## Methods

We describe the activities during the 4-year lifespan of both ARCADE HSSR and ARCADE RSDH. We accessed project meeting and discussion minutes along with periodic project reports and deliverables, and extracted the key activities and outcomes for each ARCADE work package. We summarised and discussed these. The results of the discussion are described in the following section.

## Results

### The consortia

The networked approach to capacity building was a central tenet to the ARCADE consortia. ARCADE RSDH operated through 12 universities operating within a network with expertise in research in social determinants of health or related areas, whilst ARCADE HSSR had seven partners with expertise in research of health systems (see Table 1 for a list of the partners in both projects). Both programmes were coordinated through the Karolinska Institutet (KI), based in Sweden. Initially, partners were selected on the basis of having already worked with KI and having interest and/or expertise in the subject field, after which in HSSR main partners were asked to suggest other partners to join the consortium. At each partner, principal investigators (PIs) were senior staff, supported by junior staff (postdoctoral fellows, researchers, or PhD students) in running the project.

**Table 1:** ARCADE partners

Institution (Abbreviations)	Country	ARCADE HSSR	ARCADE RSDH
Beijing Normal University (BNU)	China		x
Hanoi Medical University (HMU)	Vietnam		x
Indian Institute of Health Management Research (IIHMR)	India		x
Institute for Development Studies (IDS)	United Kingdom	x	x
Karolinska Institutet (KI)	Sweden	x	x
Makerere University (MU)	Uganda	x	
Malawi University (MA)	Malawi	x	
Muhimbili University of Health and Allied Sciences (MUHAS)	Tanzania	x	
Norwegian Knowledge Centre for Health Services (KS)	Norway	x	
Public Health Foundation of India (PHFI)	India		x
St John's Academy of Health Sciences (SJNAHS)	India		x
Stellenbosch University (SU)	South Africa	x	x
Sultan Qaboos University (SQU)	Oman		x
Tongji Medical College of HUST (TJMC)	China		x
Ujjain Charitable Trust Hospital (UCTH), Ruxmaniben Deepchand Gardi Medical College (RDGMC )	India		x
University of Tampere (UTA)	Finland		x
Zhejiang University (ZJU)	China		x

The traditionally inequitable balance of power and resources between northern and southern institutions has often led to what has been described as scientific colonialism (18). Recognising this, and taking into account the importance of allowing LMICs to take the lead in research collaborations (19), southern leadership of the consortia was an important principle for both projects. Although KI, as a northern institution, was project coordinator; most work package leaders were from southern partners and much emphasis was placed on ensuring that the project activities would take place in the south and would meet the needs of southern institutions. The intention was to build capacity and share experiences, with decreasing involvement and assistance from northern partners.

This principle was reflected in both the hub system used by the networks and also in the funding structure of the programmes. Most resources in both projects were based at two regional hub institutes: at a Chinese and Indian institute in RSDH and a South African and Ugandan Institute in HSSR. The activities and outputs in both consortia were further divided into work packages, with a consortium partner taking responsibility for each work package. The funding structure echoed the hub structure, with the partners with greater responsibility (i.e. as a hub or a work package leader) being allocated more funding than those with less responsibility.

### Consortium activities

The two consortia had five core activities in common as outlined below.

#### Needs assessment

Establishing the capacity-building needs of partner institutes was an important first goal of both ARCADEs. In ARCADE HSSR, this exercise was led by Makerere University (MU), Uganda, while in ARCADE RSDH, the exercise was led by Sultan Qaboos University, Oman (SQU). Both qualitative and quantitative methods were used to survey the available capacity for postgraduate research training at the participating institutes. Views of several different stakeholder categories were included: PhD students, supervisors, deans, faculty, administrative staff, to name but a few. The full results of the survey and qualitative methods are available elsewhere (20). Through the needs assessment process, ARCADE RSDH also developed indicators with which it was hoped that the project progress could be measured throughout its lifecycle.

Both needs assessments identified large gaps in training materials, infrastructure, and teaching capacity, similar to other studies (12, 13, 21, 22). Following this exercise, the consortia could refine the proposed list of courses identified at the proposal development stage (see Tables 2 and 3), and identify other challenges such as lack of supervision and lack of infrastructure and guidance (20). Based on these results, the consortium designed and developed relevant materials for HSSR and RSDH training, as described later.

**Table 2:** Coverage of learning modules developed – Health Systems and Services Research

Identified topical areas in proposal	Module identified for potential development in the needs assessment	Related modules developed
Quantitative methods	Epidemiology and Biostatistics	Meta-Analysis of Diagnostic Accuracy Studies
	Experimental Epidemiology	Introduction to Health Systems Research Methods
	Environmental Epidemiology	Economic Evaluation in Healthcare
	Statistical Computing and Data Management	Applied Survival Analysis
	Systematic Reviews	Randomised Controlled Trials
	Cost-Effective Analysis and Economic Evaluation	Pragmatic Randomised Controlled Trials
	Case Management	Quantitative Research Methods
	Introduction to Health Economics	
	Good Clinical and Laboratory Practice	
	Scientific Writing	
Qualitative and combined methods	Qualitative Research Methods	Practical Approaches to Qualitative Research/Qualitative Research Methods
	Ethical Conduct of Research	An Introduction to Implementation Research
		A e-book Guide to Implementation Research
Health services strengthening and programme development	Introduction to Health Services Research	Qualitative Evaluation in Health Care
	Quality of Health Models and Measures	Globalisation and Health
	Health Policy and Planning	
	Monitoring and Evaluation of Health Priorities	
Health systems	Health Systems Management and Research	Behavioural Change Communication
	Social and Behavioural Determinants of Health	Introduction to Health Systems
	Introduction to Global Health	The Challenges Faced by Health Systems in the 21st Century
		Complex Adaptive Systems
		mHealth, Health Systems and Development

**Table 3:** Coverage of learning modules developed – research on social determinants of health

Identified topical areas in proposal	Modules identified for potential development in the WP2 needs assessment	Related modules developed
Epidemiology, demography, environmental medicine, public policy and relationship, and social protection and health economics	Introduction to Epidemiology Lifecourse Epidemiology Health Economics	Principles and Methodology in Epidemiology Social Medicine
		Water and Sanitation
		Health, Environment and Development
		Medical Psychology
		Health Economics
		Climate, Society and Health
Anthropology, community-based healthcare, evaluation sciences, and health management and economics	Introduction to Sociology	Traffic Injuries
	Medical Sociology	Social Determinants and cardiovascular diseases (CVD) with Reference to India
	Inequalities in health	Gender, Women, and Health
	Social Inequality: Class, Gender, Ethnicity, Sociology of Gender	Social Determinants of Health Introductory Module
		Social Determinants of HIV
Health services strengthening and programme development	Building Research Capacity/Continuing Education: Career, Education and Life Planning, Interdisciplinary Courses	Data Processing and Analysis Improving Drug Use, especially Antibiotics Qualitative Evaluation in Health Care
	Statistics, Research and Evaluation Methods/Research Ethics: Quantitative Methods, Qualitative Methods, Research Ethics	Health Communication or Behaviour Change Communication Social Protection
Health systems		Health System Strengthening
		Policy Influence and Research Uptake
		Health and Development
		Health Policy Process in China: A Complex Adaptive Systems Perspective

#### Course materials development

One of the key outputs in both ARCADE programmes was the development and delivery of a suite of online teaching and learning materials for master's, PhD, and postdoc students. The materials and courses developed were based on their two thematic research areas (health systems and social determinants of health). The consortia used two definitions: ‘courses’ that were full courses available for teaching and ‘modules’, denoting course materials that could be used as part of teaching or as self-learning.

While writing the proposal, the consortium outlined a range of topics, which would later provide the framework for creating the online learning modules and courses. The topic list was also reviewed and refined in response to the needs assessment and later at consortium meetings as the project progressed. Tables 2 and 3 include the areas identified for development by ARCADE HSSR and ARCADE RSDH proposals, the areas identified by the needs assessment and the final complement of courses developed by the two projects.

The consortia created 25 courses and 30 modules, some of which were shared across the two programmes. ARCADE RSDH, the larger consortium developed the balance of the modules, while 11 and 14 blended learning courses were developed by ARCADE HSSR and ARCADE RSDH, respectively. The consortia experimented and tested new formats for teaching and learning which incorporated the use of digital technologies and online content.

All of the partners were expected to create their own online content, which was made available through their own institutional teaching platform and/or a central ARCADE online course repository (OCR – www.courses.arcade.project.org). The OCR was built using the open source software, Moodle. This platform was selected as it gave the consortium flexibility in design and features and because there were no ongoing cost implications. The OCR was hosted and managed by the Karolinska Institute and will continue to exist for at least 2 years beyond the end date of the project.

One of the central aims of the project was to ensure that the teaching materials developed were made freely available to researchers based in low- and middle-income settings. The online teaching materials were released under a Creative Commons 3.0 license, which allows anyone to share and adapt the content for non-commercial purposes, as long as they give appropriate credit and share the resulting material using the same license. All of the teaching materials were also made available in both large and small file sizes in order to ensure that the content is available to those accessing the material in low bandwidth settings.

#### Course delivery

As materials for specific courses became available online, the focus shifted to delivering courses and developing student capacity. Delivering ARCADE courses had several functions in the consortia: 1) testing the developed courses for their appropriateness and utility; 2) exposing ARCADE staff to e-learning methods and new educational technologies; 3) training students in skills appropriate for HSSR and SDH research; 4) collaborating across institutions; and 5) building the capacity of junior staff in teaching.

Courses were delivered in the ARCADEs either within established curricula or as freestanding courses. Courses were more readily accepted into curricula where they had previously been implemented face-to-face. As an example, the randomised controlled trials course in ARCADE HSSR was previously a face-to-face course within the master's in Clinical Epidemiology programme at Stellenbosch University (SU) in South Africa. This course was modified and adapted to become a blended learning course and was implemented at MU and KI. Partners engaged both senior staff and junior staff (postdocs or junior researchers) in developing and implementing courses, and each course or module developed engaged between one and five staff members, depending on the topic and composition. Courses could be implemented at one institute, or across partners, as in the randomised controlled trials course above.

The teaching approach could be either standalone courses (23), self-paced learning without interaction with peers or lecturers, or blended learning courses, combining both real-time instruction through the web or face-to-face with online, computer-aided instruction (16). The lecturers involved were supported centrally from KI in developing the courses and by their own e-learning and IT experts where available. Both consortia took a ‘learning by doing’ approach instead of engaging in formal training before implementing courses [see (24) in this issue]. Most lecturers also retained a constructivist approach to teaching and learning (25) that focussed on a shared construction of knowledge in real time or through online discussions instead of lecturer-driven teaching. In total, the two consortia reached 924 postgraduate students (277 in Africa and 647 in Asia), with the balance of students engaged as master's level students and fewer at the PhD level. Over half the students (507 of 924) were female.

As several courses were delivered synchronously across institutes, many of them focussed on real-time interaction and lecturing (24). Courses that were delivered through European institutions, mainly KI, were awarded credits through the European Credit Transfer System, which increased interest particularly in Africa.

#### Student mentorship, networked proposals, and grant writing

As the projects focussed on research skills capacity, and capacity building in general, postgraduate students were at the centre of both projects. These students could also have key roles in health systems or policymaking.

Both projects intended that the skills taught in courses, for example, on research methods, should be implemented by students during and after the project period, particularly in writing funding proposals. The projects also intended that staff from all partners should mentor individual students, thus building working relationships and allowing students to benefit from expertise outside their own university. Students and staff participating in research proposal writing could also attract more research funding to universities and thus create opportunities for further research and junior staff employment.

ARCADE HSSR took a systematic approach to mentoring postgraduate students, through inviting students from southern partners and mentors (senior staff or in some cases postdocs) from southern partners to two workshops, where students could have the opportunity to develop protocols for research and funding. ARCADE RSDH also adopted a number of innovative approaches to mentoring. For example, the research clinics concept, originally piloted in ARCADE HSSR, was implemented frequently in ARCADE RSDH (26). These clinics resembled journal clubs but were conducted using web conferencing technology. This concept was well received by students and seemed especially popular at the Ujjain Charitable Trust Hospital, with international participation from other Asian institutions.

Students were encouraged to write their own proposals for funding, which resulted in 18 PhD registration proposals, three postdoc and 13 master project proposals in HSSR, and 25 PhD proposals in RSDH. In addition, the consortia aimed to foster relationships between universities and to bring in funding to solidify these relationships. Thus, the outputs of both ARCADEs also included cross-institutional research proposals. In ARCADE HSSR, partners produced 18 collaborative proposals, while in ARCADE RSDH partners submitted 42 collaborative proposals. Proposals that included more than one ARCADE partner were considered collaborative. These proposals needed to have a strong HSSR focus in HSSR and an RSDH focus in ARCADE RSDH. They were sent to major international and country-based funders, and nearly 20% were funded.

Another important component of capacity building was formalised relationships between institutions. In the ARCADE HSSR project, the joint degree framework was expanded between KI and MU, to also include SU in South Africa. At the end of the project, there was a triangle of universities involved in training students: joint degrees between KI and MU, MU and SU, SU and MU, and KI and SU. While these relationships were possible in ARCADE HSSR, institutions in ARCADE RSDH aimed to develop inter-university research programmes, which would eventually lead to more formalised relationships.

#### Institutional capacity building in grants management and communications

KI, as an institute with a sound track record of attracting grants and an efficient grants office, took responsibility for building grants management capacity through their grants office. Several workshops were conducted for both Africa and Asia, and in ARCADE HSSR grants office staff worked together to present their work at conferences. Combined with this training, the project also encouraged all participants to submit joint funding applications to further develop institutional capacity building. Grants office staff from partner universities were central in supporting the funding applications submitted to major funders and collaborative proposals as described above.

The consortia intended to ensure that participating institutes could also advertise their work through communicating on social and traditional media and conferences, and with policymakers and other stakeholders For some of these activities, ARCADE partnered with another EU project, CommHERE, specialising in communicating EU projects. ARCADE also participated in the ResUpMeetUP training exchange, aiming at research uptake (27).

## Discussion

We presented an overview of the research capacity-building activities of ARCADE HSSR and ARCADE RSDH. The efforts of the consortia were comprehensive, focussing on developing the research skills, knowledge, and capabilities of young researchers in both research and in lecturing online content. As an added benefit, the funding attained allowed for building infrastructure in the participating universities.

Both the projects focussed on the southern LMIC partners – an approach previously supported by other researchers (19). The intention was therefore to foster mutually beneficial and effective relationships to achieve common objectives and to build capacity (18). This, however, was not simple in practice. To some extent, the consortia succeeded in setting the agenda together, conducting research in Africa for Africa (28) and in Asia, for Asia. Initially, however, there were challenges in getting partners to buy into the project and to take leadership. Later, in particular in ARCADE HSSR, MU and SU took leadership in implementing their work packages, training students, and attracting staff and students to the programme. Similarly, hub institutions in ARCADE RSDH took leadership.

Some of the buy-in challenges were due to the focus on e-learning being new to most partners. The increasing global demand for education (29) combined with the advancement of digital technologies and the growing reach of the Internet has created a plethora of new and varied e-learning initiatives and approaches (30), which are infrequently discussed in the context of global health research. Although strengthening health research capacity in LMICs has been on the agenda of high-profile journals such as the *Lancet* (31), this issue is discussed only sporadically, typically without reference to a particular field of study. ARCADE HSSR and ARCADE RSDH were innovative projects and could be considered a pilot, a test ground for southern and northern researchers to explore e-learning methods and contribute knowledge to this rapidly expanding field. However, this also created some challenges: e-learning was new and not the core business of most partners and enthusiasm was initially difficult to muster. The project partners were public health researchers, busy experts in their own content areas, but unfamiliar with both blended learning and the use of e-learning technologies (24). When partners recognised the potential of e-learning in improving access (32), reducing the carbon footprint by not requiring travel (33), increasing flexibility (34), increasing access for women (35), and increasing the potential of being more affordable than the sandwich model of training (36), participation increased.

The core activity in the projects was developing and implementing courses for postgraduate students. The focus on postgraduate students was important, as one of the keys to excellent research institutions is a balance of established academics with a pool of promising young scientists (37). Thus, student capacity building was seen as also being a key component of institutional capacity building. Overall, nearly 1,000 students were reached, of whom over half were female. This represents a large number of postgraduate students benefitting from the ARCADE projects, and suggests that an e-learning approach could be viable for building capacity globally. In ARCADE HSSR, more frequently than in ARCADE RSDH, courses were also delivered concurrently at several institutions. Although this also contributed to capacity building, the process had its own challenges, including matching university schedules, time zones, and staffing requirements. The institutional bureaucracies and administrative structures in some settings meant that engaging students in courses was difficult: students might not get formal credit and therefore had little motivation to attend. This was particularly the case at some Indian and Chinese institutions: Chinese students would not get credits for courses outside the university. Most Indian partners were medical schools, which did not have a formal PhD programme and could not award credits. Although challenging, the concurrent delivery of courses may have created relationships between lecturers and students across countries.

The consortia maintained a constructivist approach to teaching and learning, through attempting to keep real-time interaction within courses despite instruction being largely online. Although difficult to implement through web conferencing, particularly in areas of low bandwidth and limited infrastructure, this real-time interaction was seen as performing an important function for the consortia: training junior staff, who facilitated sessions at remote sites, in lecturing and teaching courses. This approach also suited the ethos of the project in terms of developing global health networks: students could discuss issues across sites and become familiar with the workings and structures of other institutes. For course leaders, developing and running each course across institutes required negotiating with administration for a timeslot, getting in touch with local facilitators, and familiarisation of the available infrastructure to implement the course. Students, on the contrary, could exchange their experiences and thoughts about, for example, ethical rules or research issues in real time or on online platforms. In other settings, this interaction has been seen as beneficial (38).

The consortia produced course materials for nearly 50 courses that can be used with minor adaptations to teach HSSR and RSDH. In addition to these quantifiable outcomes, exposing junior staff to these consortia and giving them experience in the field may influence their decision or ability to do research (39). The mentoring and cross-institutional research teams instituted in the projects is a promising approach to capacity building (40). Young researchers were engaged in the ARCADE consortia and were given new, alternative pathways for developing their knowledge, skills, and career that may not have existed before. Other studies have also indicated that mentored junior staff have a higher understanding of their research skills, have higher chances of having been awarded research grants, and have higher levels of career satisfaction (41).

The consortia also developed several grant proposals during the 4 years. Grants for research are increasingly important for universities (42) and also to research careers in many settings (43). The principle is ‘excellence begets excellence’ (37) where grants attract funding, which builds reputation, which attracts staff, which results in improved grant applications and greater success in obtaining funding. Successful research careers are also built on the ability to secure grants (44), and grant writing is a key skill for both PhD students and postdoctoral researchers (45). Collaborative grant writing was beneficial in fostering working relationships, but it also created interest among non-ARCADE staff in the collaboration, as they are constantly engaged in seeking research funding. Linking universities from the south with others from the south, universities from the north with those in the south, and those in the south with universities in the north has the advantage of sourcing knowledge and expertise that are not available in one's own university, for example, in disciplines such as health economics, research areas such as mHealth, or in particular, research methods.

## Conclusions

The ARCADE projects succeeded in bringing together a large number of universities in building postgraduate student capacity in the north and in the south. Each university brought a different set of skills and expertise, competences, and diverse work cultures into the consortium. These formal and informal interactions between the different actors resulted in collaborative courses, funding proposals, and research activities, which will further enhance capacity-building activities in the participating institutions. Subsequently, we hope that this will contribute towards both a global recognition of the existing capacity within southern universities and increased capacity-building activities in the fields of health systems and social determinants of health research.

## Paper context

Research capacity building is key to developing global health research. We describe two EU funded projects, the African/Asian Regional Capacity Development in Health Systems and Services Research and for Research on Social Determinants of Health (ARCADE HSSR and ARCADE RSDH) implemented from 2011–2015. Nearly 1000 postgraduate students were exposed to international networks and training, and institutional capacity was built. Similar approaches to north south and south-south networks should be developed to build research capacity globally.
